# Comprehensive whole genome sequencing-based pharmacogenomics profiling using a personalized genome interpretation workflow

**DOI:** 10.3389/fphar.2026.1804218

**Published:** 2026-06-30

**Authors:** Aikaterini Patrinou, Alexandros Kanterakis, Gerasimos Vonitsanos, Peter J. van der Spek, George P. Patrinos

**Affiliations:** 1 Department of Computer Science and Biomedical Informatics, University of Thessaly, Lamia, Greece; 2 Foundation for Research and Technology – Hellas, Institute of Computer Science, Heraklion, Crete, Greece; 3 Computer Engineering and Informatics Department, Polytechnic School, University of Patras, Patras, Greece; 4 Department of Pathology, Faculty of Medicine and Health Sciences, Erasmus University Medical Center, Clinical Bioinformatics Unit, Rotterdam, Netherlands; 5 Laboratory of Innovative Therapeutics and Personalized Medicine, Hellenic Pasteur Institute, Athens, Greece; 6 Department of Pharmacy, School of Health Sciences, University of Patras, Patras, Greece; 7 Department of Genetics and Genomics, College of Medicine and Health Sciences, United Arab Emirates University, Al-Ain, Abu Dhabi, United Arab Emirates

**Keywords:** biomarkers, clinical decision support tool, genome informatics, open access, pharmacogenes, pharmacogenomics, workflow

## Abstract

**Introduction:**

Personalized Medicine and precision therapeutics hold promise to revolutionize modern clinical practice, by improving clinical decision making and maximizing drug efficacy, while minimizing drug toxicity, based on the unique patient’s genetic profile. Clinical decision support tools aim to help clinicians to implement genome-guided therapeutics and genomic medicine with the translation of a patient’s genomic information into a clinically meaningful format. Here, we developed a personalized genome interpretation workflow, based on open-source code for facilitating the practice of precision therapeutics.

**Methods:**

Using two different previously validated pharmacogene panels, namely, the 12-pharmacogene PREPARE study panel and the 87-pharmacogene PyPGx panel, we comprehensively analyzed and reported clinically actionable, rare and novel variants, the majority of which lie at the introns and the fringe of the pharmacogenes in question.

**Results and Discussion:**

Τwo types of pharmacogenomics (PGx) reports were also produced for three members of a Greek family from data derived from whole genome sequencing. Our results demonstrate that this genome interpretation workflow enables targeted comprehensive clinical PGx assessment, with rare and novel PGx variants included in both reports for hypothesis generation. Although this overlap would not serve as validation, our workflow holds promise to facilitate implementation of PGx in the clinic using next-generation sequencing data.

## Introduction

Genomic and Personalized Medicine is gaining momentum in modern clinical practice, aiming to deliver individualized medical care that leverages information from patient’s unique clinical and genetic characteristics, as well as patient preferences, and family health history (Bick et al., 2019; Baldacchino et al., 2020). Advances in genomic technology, such as long-read sequencing, accompanied with a reciprocal reduction of genetic analysis costs further facilitate the incorporate of genome-guided diagnosis and therapeutics interventions in modern medicine (Squassina et al., 2010). Despite these major advances, existing evidence indicates that healthcare professionals have limited genomic knowledge, often being unable to provide drug dose adjustment recommendations, and require resources to guide them towards the right clinical decisions as far as diagnosis and therapeutics are concerned (Vassy et al., 2015). This is particularly important for precision therapeutics, as treating physicians from a variety of medical specialties are in need to obtain precise guidance to individualize drug dose to maximize drug efficacy and minimize drug toxicity, as almost 15% of the approved drugs by the EMA (European Medicines Agency; www.ema.europa.eu), and approximately 10% of the drugs approved by the United States Food and Drug Administration (FDA; www.fda.gov), are accompanied by pharmacogenomic recommendations (Koutsilieri et al., 2020; Shekhani et al., 2020).

Incorporation of next-generation sequencing in the post-genomic era and the reciprocal analyses of the resulting large volume of genomic data can be challenging, as the existence of rare (MAF < 1%), population-specific and, most importantly, novel, previously unidentified, variants of unknown pathogenicity, complicates the interpretation of these data effects of PGx variants on protein function. For example, it is well-established that a large number of novel and rare variants can be found in pharmacogenes, affecting the drug metabolizing enzyme function (Mizzi et al., 2014; Lauschke et al., 2024).

Clinical decision support (CDS) tools have emerged as an attractive solution to address the abovementioned barriers by facilitating the translation of a patient’s genomic information into a clinically meaningful format hence allowing smoother integration and utilization of genomic data in clinical care (Campbell et al., 2023). In other words, CDS are bioinformatics pipelines used to reconstruct a genome sequence from the sequenced reads and to then identify genomic variants when compared to a reference genome. The most crucial element and time-consuming process of CDS tools is the analysis and interpretation of these variants, which involves the investigation of the identified variants to assess their association with and/or functional implications in disease and health. Genomic variant analysis may be used to inform diagnostic, therapeutic, and reproductive decision making at the clinical level, while it can seek to understand genomic variation in the context of a single cell to even large populations at the research level.

Scientific workflows represent structured computational processes that chain together multiple analysis steps, data transformations, and computational tools to accomplish complex scientific tasks. In the context of genomics and bioinformatics, workflows orchestrate sequences of operations such as data preprocessing, alignment, variant calling, annotation, and statistical analysis, transforming raw data through a series of well-defined computational stages. These workflows are essential for managing the complexity of modern scientific computing, where analyses often involve dozens of tools, multiple data formats, and intricate dependencies between computational steps. To facilitate the development, execution, and maintenance of scientific workflows, specialized tools known as Workflow Management Systems (WMS) have been developed. These systems provide capabilities for compiling, modeling, executing, submitting to High Performance Computing (HPC) environments, monitoring, logging, and performing other essential tasks associated with scientific workflow execution. WMS tools abstract away the complexities of job scheduling, resource management, error handling, and data provenance tracking, enabling researchers to focus on the scientific logic of their analyses rather than the computational infrastructure required to execute them. Nextflow is a workflow management system that provides a rich, Groovy-based domain-specific language (DSL) for building and executing scientific computational pipelines. Nextflow has a large collection of genetic analysis tools that fit the types of data commonly used in genomics workflows, including FASTQ files (raw sequencing reads), BAM files (aligned reads), and VCF files (variant calls), making it particularly well-suited for whole genome sequencing and PGx analysis pipelines.

We have previously developed a prototype decision support tool for PGx (Lakiotaki et al., 2016) and reported a model for determining the pathogenicity of PGx variants by evaluating the *in silico* protein prediction scores with the use of machine learning (ML), and thus highlighting the PGx variants that are most likely to alter the protein function and consequently have a PGx impact (Pandi et al., 2021). This model can be used to prioritize novel variants in PGx genes and to provide an indication of their potential PGx relevance, in contrast to variant-prioritization approaches that focus mainly on general pathogenicity. Importantly, this model supports variant-level PGx interpretation and does not perform star-allele calling. Here, we report a personalized genome interpretation workflow, derived from open-source code that can be used to report the impact of known and novel PGx variants at any place in the human genome. This work is orthogonal to prior prediction models in that it can incorporate evidence from such models, perform star-allele calling, and generate a personalized PGx profile. Using this workflow, we produced a comprehensive PGx profile of three members of a family of Greek origin from whole genome sequencing data, accompanied by the corresponding drug dose recommendations.

## Methods

### Patients and DNA analysis

Total DNA, obtained from a Greek family trio with signed informed consent was isolated from peripheral blood leukocytes, using standard laboratory methods. The study was approved by the University of Patras Ethics Committee (No 14740/20-12-2022).

Subsequently, whole genome sequencing was performed using the Illumina NovaSeq 6000 platform. All samples were sequenced with a >30x coverage and with a quality call rate of >93%. The FASTQ files generated were used as input for the subsequent analysis steps, described below.

### Selection of the workflow management system

The complete analysis presented in this study has been implemented using Nextflow (https://www.nextflow.io/docs/latest/executor.html; (Di Tommaso et al., 2017)), leveraging its DSL for workflow definition, multi-executor capabilities for deployment, and containerization technologies for reproducibility. Table 1 summarizes the Nextflow workflows and underlying tools used in this study.

**TABLE 1:** Nextflow workflows and underlying tools used in this study.

Task	Tool (URL)	Workflow (URL)
Alignment	bwa-mem2 (https://github.com/bwa-mem2/bwa-mem2)	Sarek (https://nf-co.re/sarek/3.6.1/)
Variant calling	DeepVariant (https://github.com/google/deepvariant)	
Variant annotation	VEP (https://www.ensembl.org/info/docs/tools/vep/index.html)	
PGx calling	PyPGx (https://github.com/sbslee/pypgx)	PyPGx (custom)

### Pipeline architecture

**FIGURE 1:**
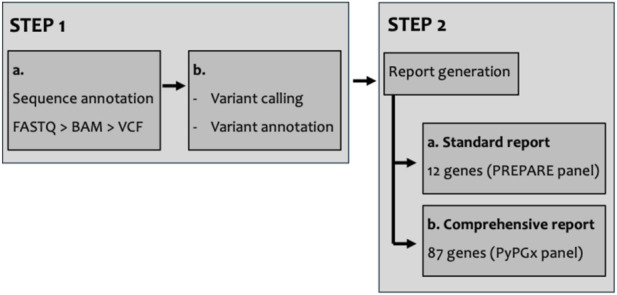
Schematic representation of the two steps that lead to the translation of raw next-generation sequencing data into two different PGx reports.

The entire pipeline is comprised of two main steps, as illustrated in Figure 1.

Step 1: Variant Calling and Annotation: Initially, paired-end FASTQ files derived from whole-genome sequencing (the output of primary analysis/basecalling) are processed through secondary analysis, including sequence alignment and variant calling, followed by tertiary analysis which consists mainly by variant annotation. This foundational stage transforms raw sequencing reads into annotated variant calls, establishing the data infrastructure required for downstream pharmacogenomic interpretation.

Subsequently, variant calling and annotation are also performed using the Sarek pipeline (Garcia et al., 2020), executed through the Nextflow workflow management system. Sarek provides a standardized, reproducible framework for orchestrating the complex series of computational steps required for germline variant analysis, including read alignment, base quality recalibration, variant calling, and variant annotation. The pipeline is configured to process each sample independently, generating one annotated VCF file per sample. This per-sample approach ensures that variant calls are not influenced by other samples in the cohort, maintaining analytical independence while allowing for downstream comparative analysis. Variant calling is performed using DeepVariant (Poplin et al., 2018), selected as the variant caller within the Sarek framework. DeepVariant employs deep neural networks trained to identify various types of genetic mutations, representing a significant advancement over traditional statistical and rule-based variant calling methods. The tool’s machine learning approach enables it to learn complex patterns in sequencing data that correlate with true genetic variants, improving accuracy particularly in challenging genomic regions such as repetitive sequences, low-complexity regions, and areas with high sequence similarity. This enhanced accuracy is critical for PGx applications, where variant calls directly inform clinical recommendations.

Following variant calling, genomic variants are then annotated using the Ensembl Variant Effect Predictor (VEP; https://useast.ensembl.org/info/docs/tools/vep/index.html; McLaren et al., 2016). VEP provides comprehensive functional annotation by predicting the molecular consequences of genetic variants, including effects on transcript structure, protein sequence, and regulatory regions. The annotation process integrates information from multiple sources to provide a holistic view of each variant’s potential biological and clinical significance. Two primary annotation resources are utilized: dbNSFP (Liu et al., 2011) version 5.2 and ClinVar (Landrum et al., 2025). dbNSFP (database of non-synonymous SNPs’ functional predictions) aggregates functional predictions from multiple algorithms and databases, providing a comprehensive assessment of variant pathogenicity and functional impact. The database includes scores from computational prediction tools such as SIFT (Sim et al., 2012), PolyPhen (Adzhubei et al., 2010), CADD (Kircher et al., 2014), DANN (Quang et al., 2015), and AlphaMissense (Cheng et al., 2023), as well as population frequency data from the 1000 Genomes Project (Auton et al., 2015) and gnomAD (Karczewski et al., 2020). This multi-dimensional annotation enables prioritization of variants based on their predicted functional consequences and population frequencies. dbNSFP is particularly valuable for PGx because it provides a unified resource that combines multiple lines of evidence for variant interpretation. Rather than querying individual prediction databases separately, dbNSFP consolidates scores from numerous algorithms, population frequency data, and clinical annotations into a single, queryable database. This integration is essential for PGx analysis, where variants must be evaluated not only for their predicted functional impact but also for their population frequencies (to distinguish common from rare potentially pathogenic variants) and their clinical associations. The comprehensive nature of dbNSFP supports both the identification of known PGx variants and the discovery of novel variants that may have PGx relevance. A comprehensive set of fields is extracted from dbNSFP to support variant interpretation is shown in Table 2.

**TABLE 2:** Fields that enable multi-dimensional variant prioritization, combining computational predictions, evolutionary conservation, population genetics, and clinical evidence to support PGx interpretation for the purpose of this study.

Fields	Fields	Purpose
Variant identifiers	rs_dbSNP, HGVSc_VEP, HGVSp_VEP	For standardized variant naming and cross-referencing
Population frequency data from the 1000 genomes project^*^	1000Gp3_EUR_AF, 1000Gp3_EAS_AF, 1000Gp3_AMR_AF	To assess variant frequency and differences across the key populations of the 1000 genomes project
gnomAD	gnomAD_exomes_AF	
Functional prediction scores	LRT_score, GERP++_RS (conservation), AlphaMissense scores and predictions, CADD scores (raw, rankscore, and phred-scaled), and DANN scores and rankscores	To provide multiple independent assessments of variant pathogenicity
Clinical annotations integrated from ClinVar	clinvar_id, clinvar_clnsig, clinvar_trait, clinvar_review, clinvar_hgvs, clinvar_var_source, clinvar_MedGen_id, clinvar_OMIM_id, clinvar_Orphanet_id	Το connect variants to established clinical phenotypes and disease associations

^*^ Considered the gold standard for population frequency data.

Also, ClinVar integration provides clinical significance annotations, including pathogenicity classifications, disease associations, and review status. This information is essential for PGx interpretation, as it connects genetic variants to established clinical phenotypes and drug response associations. ClinVar is incorporated into the Sarek pipeline through VEP’s custom annotation feature, specified via the ‘–vep_custom_args’ parameter, which adds the ClinVar VCF file as a custom annotation source and extracts key fields including clinical significance (CLNSIG), review status (CLNREVSTAT), and disease names (CLNDN). The combination of computational predictions from dbNSFP and clinical evidence from ClinVar creates a robust annotation framework that supports both discovery-oriented analysis and evidence-based clinical interpretation. The final output from Step 1 consists of annotated VCF files, with one file generated per sample. Each VCF file contains variant calls with comprehensive functional and clinical annotations, ready for downstream PGx analysis. These annotated VCF files serve as the primary input for Step 2a, while the intermediate BAM files (aligned and recalibrated reads) generated during the Sarek workflow are preserved for use in Step 2b (see below).

Step 2: PGx report generation. The second step focuses on generating a PGx report. We opted to generate two types of reports:

a. The standard PGx report, which includes variants in 12 top-tier pharmacogenes, derived from the PREPARE study (Swen et al., 2023), established by the Ubiquitous Pharmacogenomics Consortium (van der Wouden et al., 2017), which is generally considered the gold standard for preemptive PGx testing, touching upon 43 drugs in several medical specialties, such as cardiology, oncology, psychiatry, neurology, transplantation, infectious disease, and primary care. The report extends beyond the 50 PGx variants included in the PREPARE study (van der Wouden et al., 2017) to include additional known PGx variants that belong to any of the 12 PREPARE pharmacogenes, provided they have been reported by well-established PGx consortia such as the Clinical Pharmacogenetics Implementation Consortium (CPIC; (Relling and Klein, 2011)) and the Dutch Pharmacogenomics Working Group (DPWG). This expanded coverage ensures that clinically relevant variants are not overlooked, while maintaining focus on variants with established clinical guidelines.

The standard PGx report incorporates recommendations from multiple sources. It is anticipated that recommendations from different sources may be heterogenous, reflecting differences in evidence evaluation, population-specific considerations, or guideline update timeliness (Koutsilieri et al., 2020; Shekhani et al., 2020). In such cases, all recommendations are reported, with each recommendation clearly attributed to its source. This transparent approach allows clinicians to evaluate the evidence base for each recommendation and make informed decisions.

b. The comprehensive PGx report, which includes variants in 87 pharmacogenes. using PyPGx (Lee et al., 2022). This is a state-of-the-art PGx analysis tool that performs comprehensive variant detection, including single-nucleotide variants, indels, and structural variations such as gene deletions, duplications, and hybrid alleles, and integrates recommendations from multiple regulatory bodies and clinical guideline sources. This report provides a much broader PGx coverage (87 versus 12 pharmacogenes). PyPGx is executed on the full set of 87 pharmacogenes (Supplementary Table S1), providing extensive coverage beyond the 12 pharmacogenes of the standard report. PyPGx employs a support vector machine (SVM)-based multiclass classifier using a one-versus-rest strategy for each gene and each GRCh build. Each classifier is trained using copy number profiles from real next-generation sequenced samples as well as simulated data, including samples from the 1000 Genomes Project and the GeT-RM (Genetic Testing Reference Materials) program. This machine learning approach enables accurate detection of structural variants that might be missed by standard variant calling pipelines. PyPGx has been validated on the entire high-coverage whole-genome sequencing dataset from the 1000 Genomes Project (n = 2,504), demonstrating its robustness and accuracy across diverse populations.

While PyPGx can theoretically accept VCF files as input, the standard and recommended approach is to start from BAM files, as this enables PyPGx to perform its own variant calling and structural variant detection accurately. The BAM files used to generate the comprehensive PGx report are generated during Step 1 as part of the Sarek pipeline, ensuring consistency in alignment and quality control across the analysis. PyPGx is wrapped within a Nextflow workflow management system, maintaining consistency with the overall pipeline architecture and enabling reproducible execution.

The final reports assembles all identified findings into a comprehensive document that includes.

- Standard PGx report: (i) Study recommendations for the 50 tag SNPs, included in the PREPARE pharmacogene panel, (ii) rare PGx variants with their frequency and pathogenicity information in the 12 pharmacogenes, and (iii) novel PGx variants in the 12 pharmacogenes.
- Comprehensive PGx report: (i) Recommendations for the common tag SNPs, included in the 87 pharmacogenes of the PyPGx panel, (ii) rare PGx variants with their frequency and pathogenicity information in the 87 pharmacogenes mentioned above, and (iii) novel PGx variants in the 87 pharmacogenes in the PyPGx panel.

### Reference genome and output specifications

All analysis is performed using the GRCh38 (Genome Reference Consortium Human Build 38) version of the human reference genome. This reference assembly represents the current standard for genomic analysis and provides improved sequence accuracy and coverage compared to earlier assemblies. The use of GRCh38 ensures compatibility with contemporary annotation databases and clinical genomics resources, facilitating accurate variant annotation and interpretation.

### Data analysis workflow

For each sample, the following analyses are performed.

- Known PGx variants extraction and interpretation: For the standard PGx report, the genotypes for the 50 PGx variants included in the 12 PREPARE pharmacogenes are extracted from the annotated VCF file. For each variant identified, the corresponding metabolizer status is reported and the recommendation is added to the report, providing direct clinical guidance. Similarly, the comprehensive PGx report includes the specific diplotype, the metabolizer status, the clinical recommendations associated with that diplotype, and the source of the information. This comprehensive diplotype reporting enables clinicians to understand both the genetic basis of drug response and the specific clinical actions recommended.
- Extended PGx variant analysis: The workflow extracts information for additional known PGx variants that belong to the 87 pharmacogenes belonging to the comprehensive PGx report. For these variants, recommendations from the respective regulatory bodies are reported. Allele frequency information is also included, obtained either from dbNSFP annotations or from population frequency data published by Lakiotaki and coworkers (2017; (Lakiotaki et al., 2017)), providing context for the prevalence of each variant in relevant populations.
- Rare PGx variant identification: Rare PGx variants are extracted for the 12 or the 87 pharmacogenes belonging to the standard or the comprehensive report, respectively, using the 1% threshold for minor allele frequency in the European (EUR) panel of the 1000 Genomes Project. This threshold represents a balance between capturing potentially clinically significant PGx variants and avoiding common variants with uncertain functional consequences. For each rare variant identified, the report includes the allele frequency and pathogenicity classification according to ClinVar, enabling assessment of the variant’s potential clinical significance.
- Novel PGx Variant Discovery: Novel PGx variants are extracted within the 12 or the 87 pharmacogenes belonging to the standard or the comprehensive PGx report, respectively. A PGx variant is considered novel if it is not yet well characterized in existing knowledgebases and not already clearly annotated as ClinVar Pathogenic/Likely pathogenic for a PGx phenotype and are not established actionable variants in resources such as PharmGKB (Whirl-Carrillo et al., 2021) or CPIC in line with ACMG guidelines (Richards et al., 2015). This distinction is intentional: ClinVar is primarily designed for disease-gene interpretation and therefore has important limitations for PGx, where much of the relevant information is captured under germline drug-response annotations. Accordingly, and in line with ACMG principles for evidence-based variant interpretation, we use ClinVar mainly to identify pre-existing clinical classifications, while PharmGKB/CPIC are used to assess PGx actionability and established drug-gene guidance. Variants lacking established ClinVar drug-response classification and lacking PharmGKB/CPIC actionability are retained as novel PGx candidates. Novel variants represent potential discoveries that may contribute to understanding PGx variation, though their clinical significance requires careful evaluation. For each novel variant, the report includes the pathogenicity assessment from VEP, when available, providing initial guidance on potential functional impact.

### Recommendation sources

The standard and comprehensive PGx reports integrate recommendations from two categories of authoritative sources: (a) Regulatory bodies, that issue drug labeling that includes PGx information, representing official guidance on genotype-guided prescribing, and (b) Clinical guideline sources (CGS) provide evidence-based, peer-reviewed guidelines that translate PGx evidence into actionable clinical recommendations, often with more detailed dosing guidance than regulatory labeling. The sources included in each category are summarized in Table 3.

**TABLE 3:** Guidance/guideline source for the two different PGx reports generated from next-generation sequencing data (included in ClinPGx; www.clinpgx.org).

Category	Sources
Regulatory bodies	1. U.S. Food and drug administration (FDA) 2. European medicines agency (EMA) 3. Pharmaceuticals and medical devices agency of Japan (PMDA) 4. Health Canada (HCSC) 5. Swissmedic
Clinical guideline sources (CGS)	1. CPIC (Clinical pharmacogenetics implementation consortium) 2. DPWG (Dutch pharmacogenomics working group) 3. CPNDS (Canadian pharmacogenomics network for drug safety) 4. RNPGx (Réseau de pharmacogénomique)

As with the PREPARE report, conflicts between different recommendation sources are anticipated and are handled transparently: all recommendations are reported with clear source attribution (Koutsilieri et al., 2020; Shekhani et al., 2020).

### Quality control and filtering

All variant extraction and reporting is subject to quality control (QC) filtering applied to the next-generation sequencing variant calls. These QC filters ensure that only high-confidence variant calls are included in the report, minimizing false positives and maintaining analytical rigor and address variant call quality, read depth, mapping quality, and other metrics that influence confidence in variant calls.

As a first step, we apply strict quality control to minimize the inclusion of spurious calls. Variants must meet minimum depth and genotype quality thresholds (for example, per-sample depth ≥ 10–20 for SNVs and higher for indels, and genotype quality GQ ≥ 20–30), and heterozygous calls are required to have a reasonable allele balance (e.g., ∼0.25–0.75 for the alternative allele; (Pedersen et al., 2021)). Variants with strong strand bias or other poor-quality metrics (e.g., extreme FisherStrand or SOR values, problematic BaseQRankSum or ReadPosRankSum) are removed, and we generally restrict attention to variants that pass the caller’s internal FILTER criteria. These QC filters help ensure that candidates classified as “novel” are not artifacts of low coverage, sequencing noise, or misalignment (Tayeh et al., 2022).

Population allele frequency is then used as a primary filter to distinguish potentially novel or rare variants from common polymorphisms. We use multiple population resources, including gnomAD (global and subpopulation frequencies), the 1000 Genomes Project (with particular attention to the EUR panel for our predominantly European cohort), and, where available, internal cohort frequencies. As a baseline, we consider variants with MAF < 1% in the relevant population to be rare, and we prioritize as “highly novel” those variants that are absent from gnomAD/1KGP or have MAF < 0.1% (Poku-Adusei et al., 2025). Variants with MAF ≥ 5% are generally treated as common and excluded from the novel-candidate pool, while the 1%–5% range is considered low-frequency and typically receives lower priority. Population frequency information is always interpreted in the context of ancestry matching and database quality flags, as recommended in rare-variant analyses.

Next, we integrate information from clinical and PGx knowledgebases. ClinVar is used to identify variants that already have well-established clinical interpretations: variants annotated as Benign or Likely benign with multiple concordant submitters are typically deprioritized, whereas those classified as Pathogenic or Likely pathogenic for drug-response phenotypes are promoted to high-priority candidates, albeit not “novel” in a strict sense (Harrison et al., 2016). For PGx actionability, we also cross-reference PharmGKB and CPIC: variants associated with CPIC level A/B guidelines or included in known star-allele definitions are annotated as clinically actionable PGx variants and treated separately from truly novel findings (Tafazoli et al., 2020). This integration prevents us from misclassifying well-characterized benign or actionable variants as novel and focuses attention on variants that lack clear prior interpretation.

### Functional impact and *in silico* predictions

For variants that pass QC and population filters and are not already well-annotated in ClinVar or PharmGKB, we rely on functional annotation and *in silico* prediction scores available through dbNSFP. We prioritize variants with predicted protein-altering consequences—such as loss-of-function changes (stop-gain, frameshift, essential splice-site) in genes where loss of function is a plausible mechanism, and missense variants located in conserved or functionally critical domains. We use ensemble prediction metrics (e.g., REVEL (Ioannidis et al., 2016), MetaLR/MetaSVM (Dong et al., 2015), CADD) and splice-prediction scores (e.g., SpliceAI) rather than relying on single predictors, favoring variants with concordant evidence of deleteriousness (for example, REVEL > 0.5 or CADD PHRED ≥ 20, supported by multiple damaging calls; Zhou and Lauschke, 2021). Conservation metrics such as phyloP and GERP further inform prioritization; highly conserved positions with deleterious predictions and low population frequency are considered particularly compelling.

Because pharmacogenes often have gene-specific mechanisms and complex haplotype structures, general rare-variant rules must be adapted to the PGx context. Many PGx effects are defined by star-allele haplotypes and structural variants (e.g., *CYP2D6* deletions, duplications, and hybrid genes), so single-SNV-centric filters are insufficient. Wherever possible, we interpret novel variants in the context of existing star-allele frameworks and note when specialized haplotype or copy-number calling (such as that performed by PyPGx or other dedicated tools) is required (Tafazoli et al., 2020). We also acknowledge that not all loss-of-function predictions translate directly into reduced pharmacogene activity; for some genes, reduced function is driven by specific missense or promoter variants rather than generic loss-of-function alleles, so gene-specific biology must guide interpretation.

Finally, any novel PGx variants that emerge from this multi-layered filtering process are treated as candidates for careful manual review rather than as automatically actionable findings. For variants considered for clinical reporting, we recommend orthogonal confirmation (e.g., Sanger sequencing), manual curation of the literature and database entries, and classification into reporting tiers that distinguish known actionable variants, strong novel candidates, variants of uncertain pharmacogenetic significance, and variants not suitable for clinical reporting (Tayeh et al., 2022; Harrison et al., 2016; Tafazoli et al., 2020; Zhou and Lauschke, 2021; Hočevar et al., 2019). Throughout, we emphasize that minor allele frequency alone is not sufficient to infer pathogenicity; functional annotation, gene context, clinical evidence, and PGx-specific mechanisms must all be integrated before any novel variant is considered clinically meaningful. Therefore, the potential pathogenicity of novel PGx variants should be treated with caution in clinical reporting and as such these variants are not suitable for clinical decision-making but are included only for consideration by the treating phycisians and for hypothesis generation.

## Results

### DNA sequencing

DNA sequencing of the core family fulfilled the quality criteria set for the purpose of this study. In particular, for sample M/55, coverage was 28.9x (87.531 GB yield) and with Q30 = 93.81%, for sample F/54, coverage was 34.15x (105.284 GB yield) and Q30 = 94.06%, and for sample F/22, coverage was 40.95x (124.187 GB yield) and Q30 = 93.05%. FASTQ files from these samples were processed for subsequent bioinformatics analysis.

### PGx interpretation

We then used the VCF files, resulted from the conversion of the corresponding FASTQ files to produce the basic PGx report, based on the PREPARE pharmacogene panel. In particular, we identified five clinically actionable PGx variants in the M/55 sample, ten clinically actionable PGx variants in the F/54 sample and nine clinically actionable PGx variants in the F/22 sample. Based on these variants, the basic PGx report was compiled (provided in the Supplementary Material 1).

We then proceeded to analyze the additional variants, esepcially novel and rare variants in the 12 pharmacogenes included in the PREPARE panel. In particular, we identified 2168 and 35 rare and novel PGx variants, respectively in sample M/55, 2214 and 45 rare and novel PGx variants, respectively in sample F/54 and 1972 and 28 rare and novel PGx variants, respectively in sample F/22 (Table 4). The majority of the rare and novel PGx variants in the 12 PREPARE pharmacogenes are found in the intronic, upstream and downstream genomic regions, with less than 1% of the PGx variants located in genomic positions other than the previously mentioned (Table 5).

**TABLE 4:** Total number of variants identified in the 12 and 87 pharmacogene panels in the basic and comprehensive PGx report, respectively.

​	Basic PGx report			Comprehensive PGx report		
Individuals	M/55	F/54	F/22	M/55	F/54	F/22
Clinically actionable PGx variants ^*^	5	10	9	5	10	9
Rare PGx variants	2168	2214	1972	10994	11304	10826
Novel PGx variants	35	45	28	277	398	287
TOTAL	2208	2269	2009	11276	11712	11122

^*^ From the 50 PGx variant PREPARE panel. See also Supplementary Table S2 for the features of these variants.

**TABLE 5:** Total number of rare and novel variants identified in the 12-gene PREPARE panel.

Individual	M/55		F/54		M/22	
Position	Rare	Novel	Rare	Novel	Rare	Novel
Upstream	67	3	69	4	58	-
5'UTR	-	-	-	-	-	-
Missense	2	-	-	-	1	-
Synonymous	1	-	1	-	-	-
Intronic	2005	31	2051	40	1834	26
Intronic - splice donor	-	-	-	-	-	-
Intronic - splice polypyrimidine track	1	-	-	-	-	-
3'UTR	9	-	11	-	9	-
Downstream	83	1	82	1	69	2
TOTAL	2168	35	2214	45	1971	28

Two putatively pathogenic rare PGx variants were found in sample M/55. The first one was predicted by the SIFT/PolyPhen tools to have a deleterious impact on the VKORC1 protein, rendering it non-functional (Suppl. File 1). Also, the second one was predicted by the SIFT/PolyPhen tools to be tolerated by the F5 protein. In both cases, the expected phenotype was normal and as such, no drug dose adjustment recommendations were suggested. No novel variants were detected in any one of the samples analyzed.

Additionally, we then used the BAM files, resulted from the conversion of the corresponding FASTQ to produce the comprehensive PGx report, based on the PyPGx gene panel (Supplementary Table S1). Our analysis confirmed the findings resulted from the corresponding analysis of the PREPARE pharmacogene panel (Supplementary Table S4, highlighted in red). Furthermore, an additional recommendation was revealed in sample F/54 for the *IFNL3* pharmacogene (Supplementary Table S4 (highlighted in orange) and Suppl. File 2).

Subsequently, we analyzed the novel and rare variants in the 87 pharmacogenes included in the PyPGx panel. We identified 10994 and 277 rare and novel PGx variants, respectively in sample M/55, 11304 and 398 rare and novel PGx variants, respectively in sample F/54 and 10826 and 287 rare and novel PGx variants, respectively in sample F/22 (Table 4). These variants are identified within the 87 PyPGx pharmacogenes, and not across the entire genome and, as with the case of the 12 pharmacogenes of the PREPARE panel, the majority of the rare and novel PGx variants in the 87 PyPGx pharmacogenes are found in the intronic, upstream and downstream genomic regions, followed by the 3′UTR (Table 6). All identified rare and novel variants that are not clinically actionable are not included in the comprehensive PGx report to avoid confusion of treating phycisians.

**TABLE 6:** Total number of rare and novel variants identified in the 87-gene PyPGx panel.

Individual	M/55		F/54		M/22	
Position	Rare	Novel	Rare	Novel	Rare	Novel
Upstream	744	27	798	27	781	26
5'UTR	7	-	4	-	6	-
Missense	7	4	9	3	9	5
Synonymous	6	1	7	2	3	1
Intronic	9428	229	9650	351	9206	235
Intronic - splice donor	-	1	1	-	1	-
Intronic - splice polypyrimidine track	4	-	2	1	2	-
3'UTR	113	1	110	3	106	6
Downstream	685	13	723	11	712	14
TOTAL	10994	277	11304	398	10826	287

## Discussion

In this paper, we report the development of a comprehensive personalized genome interpretation workflow, based solely on open-source code that can be broadly exploited to report the impact of known, both common and rare, and novel PGx variants and to produce comprehensive PGx profiles from next-generation/whole genome sequencing data.

Our data show that the workflow performs really well as far as identification of PGx variants in both pharmacogene panels is concerned, based on which the standard and comprehensive PGx reports are generated. Also, our findings highlight that additional rare and novel variants are identified. For the standard PGx report, we did not identify novel variants with functional impact on the enzymes. We identified though rare variants with functional implications but the overall genotypes did not lead to any recommendation for adjusting drug dose. As far as the comprehensive PGx report is concerned, we identified a large number of novel and rare PGx variants. Very few of them though, all of which being missense variants, had functional implications on the respective enzyme (Supplementary Table S3), and did not lead to any recommendation for drug dose amendment.

We opted to include the PREPARE 12-pharmacogene panel as our primary reporting outcome. First of all, the PREPARE study has resulted into well-defined functional consequences and a reciprocal measurable bedside benefit (30% reduction of ADRs in several European populations with self-declared European, Mediterranean, or Middle Eastern ancestry; Swen et al., 2023) and a demonstrated cost-effectiveness when deployed pre-emptively (Fragoulakis et al., 2026). By focusing on high-prevalence actionable alleles (95% of Europeans carry at least one actionable PGx allele; Swen et al., 2023), the PREPARE panel maximizes yield even in small experimental cohorts while avoiding the interpretive uncertainty inherent to rare variants. Also the 12-pharmacogene PREPARE genotype-guided recommendations is considered the gold standard for preemptive PGx testing, touching upon 43 drugs in several medical specialties, such as cardiology, oncology, psychiatry, neurology, transplantation, infectious disease, and primary care. This contrasts with many commercial reports that focus on single ancestries, single or very few medical specialties, or self-reported phenotypes. Collectively, these attributes make reports based on the 12-pharmacogene PREPARE panel more interoperable in different healthcare care settings, more useful to regulators and payers, and better aligned with the implementation challenges faced by modern precision-medicine clinics.

There is an overlap between the two reporting approaches, where the 87 PyPGx pharmacogenes analyzed in Step 2b include all 12 PREPARE pharmacogenes of Step 2a and some redundant analysis, particularly in the extraction of rare and novel variants. However, the two steps serve complementary roles with distinct clinical focuses: Step 2a focuses specifically on the PREPARE-based recommendations and provides a direct implementation of the PREPARE study framework, with a primary emphasis on reducing Adverse Drug Reactions (ADRs) and addressing drug toxicity. The standard PGx report is designed to identify PGx variants that predispose patients to ADRs, enabling preventive interventions that minimize toxicity from drugs with genome-guided clinically actionable recommendations.

The essence of adding a second type of report (the comprehensive PGx report; Step 2b) is to manage other effects that can be addressed through PGx analysis, such as drug efficacy. While the standard PGx report prioritizes safety and toxicity prevention, the comprehensive PGx report provides broader PGx coverage that addresses both safety concerns and efficacy optimization. This broader scope enables identification of genetic factors that may lead to subtherapeutic drug levels, treatment failure, or poor response, complementing the toxicity-focused standard PGx report. Additionally, Step 2b could serve as a confirmatory step for the variants detected in Step 2a and as comparison across multiple guideline sources, and its inclusion of structural variant detection through PyPGx provides information that may not be captured in the VCF-based analysis of Step 2a, particularly for complex genes like *CYP2D6*, or VCF files derived from short-read sequencing instuments (Samarasinghe et al., 2026). Also, the comprehensive nature of the PyPGx 87-pharmacogene panel increases the likelihood of identifying novel variants that may contribute to understanding PGx diversity. Indeed, our findings show that the recommendations derived from the standard PGx report are confirmed by the comprehensive PGx report (Supplementary Table S4) and got also enriched for sample F/54 by an additional recommendation (Suppl. File 2).

Also, there are some differences in the recommendation sources used for reporting. Generation of the standard and comprehensive PGx reports utilize different starting points, reflecting the input requirements for each analytical approach. In other words, the standard report utilizes the annotated VCF files generated in Step 1b, which contain variant calls with comprehensive functional annotations. This VCF-based approach is well-suited for targeted variant extraction and interpretation based on known PGx variants. On the contrary, the process for the generation of the comprehensive PGx report begins with BAM files, generated in Step 1a. While PyPGx can accept VCF files as input, starting from BAM files is the standard and recommended approach, as it enables PyPGx to perform its own variant calling and structural variant detection with superior accuracy. The BAM-based approach allows PyPGx to leverage read-level information that may be lost in VCF format, enabling more accurate detection of complex variants, structural rearrangements, and copy number variations. All three file types (VCF, BAM, and FASTQ) are produced as outputs from Step 1, ensuring that both analytical approaches work from the same underlying sequencing data, maintaining consistency while allowing each step to use the most appropriate data format for its specific analytical requirements.

Our study has some limitations. First of all, our analysis did not reveal novel or rare variants that would have a functional impact on the respective enzymes, most likely due to the limited number of individuals analyzed. A much larger patient cohort would be needed to further demonstrate the usefulness of this tool, perhaps nicely aligning with the PGx activities of the Genome of Europe project (Work Package 6; https://genomeofeurope.eu). However, our workflow has demonstrated its ability to identify rare and novel variants and as such, although the work reported in this manuscript can be considered a proof-of-concept study, it is expected to perform equally well and tangibly in larger patient cohorts, both at an individual and aggregated level (experimental work in progress). Also, PGx variants, unlike classical genetic variants, are not immediately suitable for clinical implementation without undergoing dedicated pharmacokinetic studies, population analyses, clinical trials, and other functional validation steps required to establish evidence-based recommendations. Similarly, the use of the dbNSFP prediction tools is yet another limitation. In particular, most prediction tools (including dbNSFP) are based on single-variant, disease-oriented models and do not account for drug-specific, context-dependent factors such as substrate specificity, enzyme induction, or tissue expression. Moreover, PGx interpretation mostly depends on haplotypes and less frequently on structural variation. Therefore, these tools are not designed to capture clinically relevant PGx outcomes such as drug metabolism, toxicity, or therapeutic efficacy. As such, their applicability to PGx is not well documented and hence these tools should be used cautiously and never as primary evidence for PGx interpretation. Furthermore, while the PGx report is currently manually generated and the recommendations manually retrieved from ClinPGx, we are in the process of automating this process to retrieve the various recommendations directly from the regulatory bodies and research consortia (Table 3). Another important limitation is that it is based on BAM files derived from short reads. As a result, to perform diplotype calling, PyPGx relies on statistical phasing using the Beagle tool (Browning et al., 2021). Currently, we use the 1KGP panel for phasing, which is the default option in PyPGx, although PyPGx also supports alternative reference panels. Statistical phasing may introduce errors that can result in mis-haplotyping, especially for (a) rare variants, (b) variants that are poorly represented in the reference panel, or (c) highly complex structural variations, such as those commonly observed in CYP2D6. Although PyPGx provides alternative diplotype calls in uncertain cases and reports calling confidence, we cannot exclude the possibility of errors that could lead to invalid recommendations. We expect that the incorporation of long-read sequencing (Samarasinghe et al., 2026) and broader reference panels that include more diverse populations will significantly reduce this source of error. Lastly, we did not perform validation and reproducibility assessments of our workflow by applying the same pipeline to reference PGx materials and comparing results and did not perform external validation using another PGx translational tool. In the latter case, this was unavoidable as there is no such tool to be considered as gold standard for the annotation of PGx varisnts from NGS data to compare against our own workflow. Nevertheless, the fact that we have found the same variants in the 12 PREPARE pharmacogenes using the same pipeline in the 87 PyPGx pharmacogene panel could be considered as confirmation for the accuracy of variant calling in Step 2a.

The results from this study focus specifically on PGx analysis and does not provide a comprehensive clinicogenomic assessment for the purpose of inherited disease discovery. The analytical pipeline presented here is designed only to identify PGx variants, some of which are clinically actionable, which generate clinical recommendations for drug dosing and selection, rather than to perform genome-wide variant interpretation for rare disease diagnosis or carrier screening. The rationale and design of this workflow allows to complete a more comprehensive clinicogenomic analysis given that appropriate computational resources are available, such as the Orphanet database for rare diseases and the Online Mendelian Inheritance in Man (OMIM; www.omim.org) for disease-gene associations. We are currently working to integrate these additional analyses into the existing tool that would transform the current PGx-focused pipeline into a clinicogenomic assessment platform, enabling both PGx as well as clinical genetic recommendations to be issued. Such expansion though requires substantial additional computational resources, extended analysis time, and expertise in clinical variant interpretation, which were beyond the scope of the current study.

## Conclusion

The 2-step analytical pipeline described herein, based entirely on open-source tools, provides a comprehensive framework for PGx analysis, progressing from raw whole genome sequencing data through variant calling and annotation, then branching into two complementary reporting strategies touching upon 12 and 87 pharmacogenes, respectively, providing targeted, evidence-based recommendations, using state-of-the-art analysis tools and integrating recommendations from multiple regulatory and guideline sources that are derived from the ClinPGx database. Together, this genome interpretation workflow enables targeted comprehensive clinical PGx assessment, and holds promise to facilitate clinical implementation of PGx.

## Data Availability

The data presented in the study are deposited in the ZENODO and GitHub repositories (accession number 20808836; https://zenodo.org/records/20808836; https://github.com/kantale/prepare_eval; DOI: 10.5281/zenodo.20808835).
