# Supplementary material for: Comprehensive whole genome sequencing-based pharmacogenomics profiling using a personalized genome interpretation workflow
Source: Front Pharmacol. 2026 Jun 30;17:1804218. doi: 10.3389/fphar.2026.1804218 (PMC13365265; doi:10.3389/fphar.2026.1804218)
Supplement: Supplementary file 1 [file DataSheet1.pdf]

Supplementary Information

Comprehensive whole genome sequencing-based pharmacogenomics profiling using a personalized genome interpretation workflow

Aikaterini Patrinou [1,#], Alexandros Kanterakis [2,#], Gerasimos Vonitsanos [3], Peter J. van der Spek [4], George P. Patrinos [4,5,6,7,*]

[1] University of Thessaly, Department of Computer Science and Biomedical Informatics, Lamia, Greece

[2] Foundation for Research and Technology – Hellas, Institute of Computer Science, Heraklion, Crete, Greece

[3] University of Patras, Polytechnic School, Computer Engineering and Informatics Department, Patras, Greece

[4] Erasmus University Medical Center, Faculty of Medicine and Health Sciences, Department of Pathology, Clinical Bioinformatics Unit, Rotterdam, the Netherlands

[5] Hellenic Pasteur Institute, Laboratory of Innovative Therapeutics and Personalized Medicine, Athens, Greece

[6] University of Patras, School of Health Sciences, Department of Pharmacy, Patras, Greece

[7] United Arab Emirates University, College of Medicine and Health Sciences, Department of Genetics and Genomics, Al-Ain, Abu Dhabi, United Arab Emirates

[#] These authors contributed equally to this work

Supplementary Table 1. The 87 pharmacogenes reported from PyPGx. The 12 pharmacogenes included in the standard report are indicated in boldface.

Gene Symbol	Main PGx Function
ABCB1	**Drug transporter (P-glycoprotein); affects absorption and efflux of many drugs including chemotherapeutics, immunosuppressants, and cardiac drugs**
ABCG2	Drug transporter (BCRP); affects bioavailability and tissue distribution of drugs including statins, antineoplastics, and uric acid lowering agents
ACYP2	Acylphosphatase; associated with statin-induced myopathy risk
ADRA2A	Alpha-2A adrenergic receptor; affects response to antidepressants and antipsychotics
ADRB2	Beta-2 adrenergic receptor; affects bronchodilator response in asthma treatment
ANKK1	Ankyrin repeat and kinase domain containing 1; associated with antipsychotic response
APOE	Apolipoprotein E; affects statin response and Alzheimer's disease treatment
ATM	Ataxia telangiectasia mutated; affects response to radiation therapy and certain chemotherapeutics
BCHE	Butyrylcholinesterase; affects metabolism of succinylcholine and other ester drugs
BDNF	Brain-derived neurotrophic factor; associated with antidepressant response
CACNA1S	Calcium channel alpha-1S subunit; affects susceptibility to malignant hyperthermia with volatile anesthetics
CFTR	Cystic fibrosis transmembrane conductance regulator; affects response to CFTR modulators (ivacaftor, lumacaftor)

COMT	Catechol-O-methyltransferase; affects metabolism of catecholamines and response to levodopa and antipsychotics
CYP1A1	Cytochrome P450 1A1; Phase I metabolism of polycyclic aromatic hydrocarbons and some drugs
CYP1A2	Cytochrome P450 1A2; Phase I metabolism of caffeine, clozapine, theophylline, and other drugs
CYP1B1	Cytochrome P450 1B1; Phase I metabolism of estrogens and some chemotherapeutics
CYP2A6 / CYP2A7	Cytochrome P450 2A6/2A7; Phase I metabolism of nicotine, coumarin, and some anesthetics
CYP2A13	Cytochrome P450 2A13; Phase I metabolism in respiratory tract, affects nicotine metabolism
CYP2B6 / CYP2B7	Cytochrome P450 2B6/2B7; Phase I metabolism of efavirenz, bupropion, cyclophosphamide, and methadone
CYP2C8	Cytochrome P450 2C8; Phase I metabolism of paclitaxel, repaglinide, and some statins
CYP2C9	**Cytochrome P450 2C9; Phase I metabolism of warfarin, phenytoin, NSAIDs, and sulfonylureas**
CYP2C19	**Cytochrome P450 2C19; Phase I metabolism of clopidogrel, proton pump inhibitors, and antidepressants**
CYP2D6 / CYP2D7	**Cytochrome P450 2D6/2D7; Phase I metabolism of many antidepressants, antipsychotics, beta-blockers, and opioids**
CYP2E1	Cytochrome P450 2E1; Phase I metabolism of ethanol, acetaminophen, and some anesthetics
CYP2F1	Cytochrome P450 2F1; Phase I metabolism in lung tissue, affects toxicity of some inhaled compounds

CYP2J2	Cytochrome P450 2J2; Phase I metabolism of arachidonic acid and some drugs including ebastine
CYP2R1	Cytochrome P450 2R1; Vitamin D 25-hydroxylase; affects vitamin D metabolism
CYP2S1	Cytochrome P450 2S1; Phase I metabolism in extrahepatic tissues, affects drug activation
CYP2W1	Cytochrome P450 2W1; Phase I metabolism in colon, affects activation of some prodrugs
CYP3A4	Cytochrome P450 3A4; Phase I metabolism of many drugs including statins, immunosuppressants, and chemotherapeutics
CYP3A5	**Cytochrome P450 3A5; Phase I metabolism of tacrolimus, midazolam, and other CYP3A substrates**
CYP3A7	Cytochrome P450 3A7; Phase I metabolism primarily in fetal liver, affects drug metabolism in neonates
CYP3A43	Cytochrome P450 3A43; Phase I metabolism, minor role in drug metabolism
CYP4A11	Cytochrome P450 4A11; Fatty acid omega-hydroxylase, affects metabolism of some drugs
CYP4A22	Cytochrome P450 4A22; Fatty acid metabolism, limited drug metabolism role
CYP4B1	Cytochrome P450 4B1; Phase I metabolism in lung, affects activation of some toxins
CYP4F2	Cytochrome P450 4F2; Vitamin K metabolism; affects warfarin dosing requirements
CYP17A1	Cytochrome P450 17A1; Steroid biosynthesis; affects response to hormone therapy

CYP19A1	Cytochrome P450 19A1 (Aromatase); Estrogen synthesis; affects aromatase inhibitor response
CYP26A1	Cytochrome P450 26A1; Retinoic acid metabolism; affects retinoid therapy
DBH	Dopamine beta-hydroxylase; Catecholamine synthesis; affects response to antipsychotics and antidepressants
DPYD	**Dihydropyrimidine dehydrogenase; Pyrimidine metabolism; affects 5-fluorouracil and capecitabine toxicity**
DRD2	Dopamine D2 receptor; Affects response to antipsychotics and antiemetics
F2	Coagulation factor II (Prothrombin); Affects thrombosis risk and anticoagulant response
F5	**Coagulation factor V (Factor V Leiden); Affects thrombosis risk and oral contraceptive safety**
G6PD	Glucose-6-phosphate dehydrogenase; Affects hemolytic risk with primaquine, rasburicase, and other oxidizing drugs
GRIK1	Glutamate receptor ionotropic kainate 1; Affects response to some neurological treatments
GRIK4	Glutamate receptor ionotropic kainate 4; Associated with antidepressant response
GRIN2B	Glutamate receptor ionotropic NMDA 2B; Affects response to NMDA receptor modulators
GSTM1	Glutathione S-transferase mu 1; Phase II conjugation; affects detoxification of chemotherapeutics and environmental toxins
GSTP1	Glutathione S-transferase pi 1; Phase II conjugation; affects response to platinum-based chemotherapeutics
GSTT1	Glutathione S-transferase theta 1; Phase II conjugation; affects detoxification of various drugs and toxins

HTR1A	Serotonin receptor 1A; Affects response to antidepressants and anxiolytics
HTR2A	Serotonin receptor 2A; Affects response to antidepressants and antipsychotics
IFNL3	Interferon lambda 3; Affects response to peginterferon-based hepatitis C treatment
ITGB3	Integrin beta 3; Affects platelet aggregation and response to antiplatelet drugs
ITPA	Inosine triphosphatase; Affects ribavirin-induced hemolytic anemia risk
MTHFR	Methylenetetrahydrofolate reductase; Folate metabolism; affects methotrexate toxicity and homocysteine levels
NAT1	N-acetyltransferase 1; Phase II acetylation; affects metabolism of aromatic amines and some drugs
NAT2	N-acetyltransferase 2; Phase II acetylation; affects metabolism of isoniazid, hydralazine, and procainamide
NUDT15	**Nudix hydrolase 15; Affects thiopurine (azathioprine, mercaptopurine, thioguanine) toxicity**
OPRK1	Opioid receptor kappa 1; Affects response to opioid analgesics
OPRM1	Opioid receptor mu 1; Affects response to opioid analgesics and risk of opioid dependence
POR	Cytochrome P450 oxidoreductase; Electron donor for CYP enzymes; affects multiple CYP-mediated drug metabolism
PTGIS	Prostacyclin synthase; Affects response to prostacyclin analogs
RARG	Retinoic acid receptor gamma; Affects response to retinoid therapy
RYR1	Ryanodine receptor 1; Affects susceptibility to malignant hyperthermia with volatile anesthetics and succinylcholine

SLC6A4	Serotonin transporter; Affects response to selective serotonin reuptake inhibitors (SSRIs)
SLC15A2	Solute carrier family 15 member 2 (PEPT2); Affects transport of peptide-like drugs including beta-lactam antibiotics
SLC22A2	Solute carrier family 22 member 2 (OCT2); Affects renal excretion of metformin and other organic cations
SLC28A3	Solute carrier family 28 member 3 (CNT3); Affects transport of nucleoside analogs
SLC47A2	Solute carrier family 47 member 2 (MATE2-K); Affects renal excretion of metformin and other organic cations
SLCO1B1	**Solute carrier organic anion transporter 1B1; Affects hepatic uptake of statins and other drugs, influences statin-induced myopathy risk**
SLCO1B3	Solute carrier organic anion transporter 1B3; Affects hepatic uptake of drugs including statins and chemotherapeutics
SLCO2B1	Solute carrier organic anion transporter 2B1; Affects intestinal absorption of drugs including statins
SULT1A1	Sulfotransferase 1A1; Phase II sulfation; affects metabolism of estrogens, tamoxifen, and other drugs
TBXAS1	Thromboxane A synthase 1; Affects platelet function and response to antiplatelet drugs
TPMT	**Thiopurine S-methyltransferase; Phase II methylation; affects thiopurine (azathioprine, mercaptopurine, thioguanine) metabolism and toxicity**
UGT1A1	**UDP-glucuronosyltransferase 1A1; Phase II glucuronidation; affects metabolism of irinotecan, bilirubin, and other drugs**
UGT1A4	UDP-glucuronosyltransferase 1A4; Phase II glucuronidation; affects metabolism of lamotrigine and other drugs

UGT1A6	UDP-glucuronosyltransferase 1A6; Phase II glucuronidation; affects metabolism of serotonin and some drugs
UGT2B7	UDP-glucuronosyltransferase 2B7; Phase II glucuronidation; affects metabolism of opioids, NSAIDs, and other drugs
UGT2B15	UDP-glucuronosyltransferase 2B15; Phase II glucuronidation; affects metabolism of androgens and some drugs
UGT2B17	UDP-glucuronosyltransferase 2B17; Phase II glucuronidation; affects metabolism of androgens and some drugs
VKORC1	**Vitamin K epoxide reductase complex subunit 1; Affects warfarin dosing requirements and anticoagulation response**
XPC	Xeroderma pigmentosum complementation group C; DNA repair; affects response to platinum-based chemotherapeutics

Supplementary Table 2. Clinically actionable variants in the 12 pharmacogenes of the PREPARE panel, identified in the trio analyzed within the scope of this study.

M/55

Position	Gene	Ref	Alt	Genotype	Consequence	Feature	Allele	Effect on Protein	Functional Status
ch2:233760233	*UGT1A1*	$(TA)_6$	$(TA)_7$	$(TA)_6/(TA)_7$	upstream_gene_variant	NM_000463.3	*28	Regulatory	Decreased
ch7:99672916	*CYP3A5*	T	C	C/C	intron_variant	NM_000777.5	*3	Splice defect	Inactive
ch10:94761900	*CYP2C19*	C	T	C/T	upstream_gene_variant	NM_000769.4	*17	Regulatory	Increased
ch15:74749576	*CYP1A2*	C	A	C/A	intron_variant	NM_000761.5	*1F	Regulatory	Higher inducibility
ch19:41016810	*CYP2B6*	C	T	C/T	missense_variant	NM_000767.5	T	p.R487C	Decreased

F/54

Position	Gene	Ref	Alt	Genotype	Consequence	Feature	Allele	Effect on Protein	Functional Status
ch2:233760233	*UGT1A1*	$(TA)_6$	$(TA)_7$	$(TA)_6/(TA)_7$	upstream_gene_variant	NM_000463.3	*28	Regulatory	Decreased
ch7:99672916	*CYP3A5*	T	C	C/C	intron_variant	NM_000777.5	*3	Splice defect	Inactive
ch10:94761900	*CYP2C19*	C	T	C/T	upstream_gene_variant	NM_000769.4	*17	Regulatory	Increased
ch10:94942290	*CYP2C9*	C	T	C/T	missense_variant	NM_000771.4	*2	p.R144C	Decreased
ch12:21178615	*SLCO1B1*	T	C	T/C	missense_variant	NM_006446.5	*5/*15/*17	p.V174A	Decreased
ch15:74749576	*CYP1A2*	C	A	A/A	intron_variant	NM_000761.5	*1F	Regulatory	Higher inducibility
ch19:41006936	*CYP2B6*	G	T	G/T	missense_variant	NM_000767.5	*6	p.Q172H	Decreased or inactive
ch19:41009358	*CYP2B6*	A	G	A/G	missense_variant	NM_000767.5	*16	p.K252R	Decreased or inactive
ch22:42128945	*CYP2D6*	C	T	C/T		NM_000106.6	*4	Splicing defect	Inactive
ch22:42130692	*CYP2D6*	G	A	G/A	missense_variant	NM_000106.6	*10	p.P34S	Decreased

F/22

Position	Gene	Ref	Alt	Genotype	Consequence	Feature	Allele	Effect on Protein	Functional Status
ch2:233760233	*UGT1A1*	$(TA)_6$	$(TA)_7$	$(TA)_6/(TA)_7$	upstream_gene_variant	NM_000463.3	*28	Regulatory	Decreased
ch7:99672916	*CYP3A5*	T	C	C/C	intron_variant	NM_000777.5	*3	Splice defect	Inactive
ch10:94761900	*CYP2C19*	C	T	C/T	upstream_gene_variant	NM_000769.4	*17	Regulatory	Increased
ch12:21178615	*SLCO1B1*	T	C	T/C	missense_variant	NM_006446.5	*5/*15/*17	p.V174A	Decreased
ch15:74749576	*CYP1A2*	C	A	A/A	intron_variant	NM_000761.5	*1F	Regulatory	Higher inducibility
ch19:41006936	*CYP2B6*	G	T	G/T	missense_variant	NM_000767.5	*6	p.Q172H	Decreased or inactive
ch19:41009358	*CYP2B6*	A	G	A/G	missense_variant	NM_000767.5	*16	p.K252R	Decreased or inactive
ch22:42128945	*CYP2D6*	C	T	C/T		NM_000106.6	*4	Splicing defect	Inactive
ch22:42130692	*CYP2D6*	G	A	G/A	missense_variant	NM_000106.6	*10	p.P34S	Decreased

Supplementary Table 3. Novel variants found in genes belonging to the PyPGx 87-pharmacogene panel, with a pathogenic impact identified in the trio analyzed within the scope of this study. All variants are missense and their functional impact is shown in the last two columns of the table.

M/55

Position	Ref	Alt	Genotype	Feature	Gene	CDS	HGVSc	HGVSp	SIFT	PolyPhen
ch2:38075195	G	C	G/C	NM_000104.4	*CYP1B1*	194	c.194C>G	NP_000095.2:p.A65G	Deleterious (0)	Benign (0.323)
ch19:40849901	A	T	A/T	NM_000762.6	*CYP2A6*	260	c.260T>A	NP_000753.3:p.V87D	Deleterious (0)	Probably_damaging (0.934)
ch20:49524060	G	C	G/C	NM_000961.4	*PTGIS*	853	c.853C>G	NP_000952.1:p.Q285E	Deleterious (0)	Possibly_damaging (0.755)
ch19:38585973	C	A	C/A	NM_000540.3	*RYR1*	14839	c.14839C>A	NP_000531.2:p.Q4947K		Unknown (0)

F/54

Position	Ref	Alt	Genotype	Feature	*Gene*	CDS	HGVSc	HGVSp	SIFT	PolyPhen
ch1:109689072	C	G	C/G	NM_000561.4	*GSTM1*	202	c.202C>G	NP_000552.2:p.H68D	Deleterious_low_confidence (0)	Probably_damaging (0.979)
ch19:44908924	G	C	G/C	NM_000041.4	*APOE*	628	c.628G>C	NP_000032.1:p.A210P	Deleterious (0.02)	

F/22

Position	Ref	Alt	Genotype	Feature	Gene	CDS	HGVSc	HGVSp	SIFT	PolyPhen
ch2:38074979	T	G	T/G	NM_000104.4	*CYP1B1*	410	c.410A>C	NP_000095.2:p.T137S	Deleterious (0.05)	Probably_damaging (0.948)
ch7:75985143	A	T	A/T	NM_001395413.1	*POR*	1325	c.1325A>T	NP_001382342.1:p.D442V	Deleterious (0)	Possibly_damaging (0.581)
ch20:49524072	G	C	G/C	NM_000961.4	*PTGIS*	841	c.841C>G	NP_000952.1:p.L281V	Deleterious (0)	Probably_damaging (0.967)

Supplementary Table 4. Output of the PyPGx report for the 3 family members of the trio analyzed. Recommendations also produced from the analysis of the 12 pharmacogene PREPARE panel are highlighted in red and not included in the comprehensive PGx report. Additional recommendations from the analysis of the other pharmacogenes of the 87 PyPGx panel are highlighted in orange and are available in the comprehensive PGx report (Source: www.clinpgx.org). All identified rare and novel variants that are not clinically actionable are not included in the comprehensive PGx report to avoid confusion of treating phycisians.

M/55

Gene	Genotype	Phenotype
UGT2B7	*1/*2	Indeterminate
RARG	Reference/Reference	Indeterminate
CYP26A1	*1/*1	Indeterminate
CYP2S1	*1/*3	Indeterminate
OPRM1	Reference/Reference	Indeterminate
BDNF	Reference/Reference	Indeterminate
NAT2	*5/*5	Indeterminate
GSTP1	*B/*B	Indeterminate
SLC22A2	*1/*2	Indeterminate
CYP2C19	*1/*17	Rapid Metabolizer
SLCO1B3	rs7311358/rs7311358	Indeterminate
CYP2A13	*1/*1	Indeterminate
NUDT15	*1/*1	Normal Metabolizer
CYP2A6	*1/*1	Indeterminate
CYP17A1	Reference/Reference	Indeterminate
SLC28A3	Reference/Reference	Indeterminate
APOE	Reference/Reference	Indeterminate
CYP19A1	*1/*4	Indeterminate
IFNL3	Reference/Reference	Favorable Response
RYR1	Reference/Reference	Uncertain Susceptibility

SLC47A2	Reference/rs12943590	Indeterminate
CYP2B6	*1/*5	Normal Metabolizer
ATM	Reference/rs11212617	Indeterminate
ITGB3	Reference/Reference	Indeterminate
DPYD	Reference/Reference	Normal Metabolizer
ANKK1	Reference/Taq1A	Indeterminate
UGT1A1	*80/*80+*28	Indeterminate
CYP2F1	*5/*5	Indeterminate
DBH	Reference/rs1611115	Indeterminate
UGT1A6	Reference/Reference	Indeterminate
MTHFR	rs1801131/rs1801133	Indeterminate
F2	Reference/G20210A	Indeterminate
ABCG2	Reference/Reference	Normal Function
F5	Reference/Reference	Favorable Response
ADRA2A	Reference/rs1800544	Indeterminate
CYP4F2	*1/*1	Indeterminate
BCHE	Reference/Reference	Indeterminate
ABCB1	*1/*1	Indeterminate
CYP2E1	*1/*1	Indeterminate
CYP2J2	*1/*1	Indeterminate
CFTR	Reference/Reference	Indeterminate
GRIK4	Reference/Reference	Indeterminate
ITPA	Reference/Reference	Indeterminate
SLC6A4	Reference/Reference	Indeterminate
CYP1A1	*1/*1	Indeterminate
CYP3A5	*3/*3	Poor Metabolizer
HTR1A	Reference/Reference	Indeterminate
CYP2W1	*1/*1	Indeterminate
DRD2	Reference/Reference	Indeterminate
CYP1B1	*1/*2	Indeterminate
CYP3A4	*1/*1	Indeterminate
ADRB2	Reference/rs1042713	Indeterminate

HTR2A	rs6311/rs7997012	Indeterminate
CYP4A11	*1/*1	Indeterminate
ACYP2	Reference/Reference	Indeterminate
POR	*28/*28	Indeterminate
OPRK1	Reference/rs6473797	Indeterminate
NAT1	*4/*4	Indeterminate
CYP3A43	*1/*1	Indeterminate
SLC15A2	*2/*2	Indeterminate
VKORC1	Reference/Reference	Indeterminate
UGT2B15	*2/*4	Indeterminate
CYP4B1	*1/*2	Indeterminate
CYP2D6	*1/*2	Normal Metabolizer
GSTM1	*0/*0	Indeterminate
GRIN2B	rs2058878/rs2058878	Indeterminate
G6PD	B (reference)	G6PD Normal
SLCO1B1	*1/*14	Normal Function
SULT1A1	*1x2/*2	Indeterminate
PTGIS	*1/*1	Indeterminate
TBXAS1	*1/*1	Indeterminate
CYP2C8	*1/*1	Indeterminate
UGT1A4	*1/*1	Indeterminate
CYP2C9	*1/*1	Normal Metabolizer
CACNA1S	Reference/Reference	Uncertain Susceptibility
XPC	Reference/rs2228001	Indeterminate
CYP2R1	*1/*1	Indeterminate
CYP4A22	*1/*1	Indeterminate
CYP1A2	*1A/*1F	Indeterminate
TPMT	*1/*1	Normal Metabolizer
SLCO2B1	*1/*1	Indeterminate
CYP3A7	*1/*1	Indeterminate
GRIK1	Reference/rs2832407	Indeterminate
COMT	rs174675/rs9606186	Indeterminate

F/54

**Gene**	**Genotype**	**Phenotype**
UGT2B7	*1/*2	Indeterminate
RARG	Reference/Reference	Indeterminate
CYP26A1	*1/*1	Indeterminate
CYP2S1	*1/*1	Indeterminate
OPRM1	Reference/rs1799971	Indeterminate
BDNF	Reference/Reference	Indeterminate
NAT2	*5/*6	Indeterminate
GSTP1	*B/*B	Indeterminate
SLC22A2	*2/*3	Indeterminate
CYP2C19	*1/*17	Rapid Metabolizer
SLCO1B3	rs7311358/rs7311358	Indeterminate
CYP2A13	*1/*1	Indeterminate
NUDT15	*1/*1	Normal Metabolizer
CYP2A6	*1/*2	Indeterminate
CYP17A1	Reference/Reference	Indeterminate
SLC28A3	Reference/Reference	Indeterminate
APOE	Reference/Reference	Indeterminate
CYP19A1	*1/*1	Indeterminate
IFNL3	**Reference/rs12979860**	**Unfavorable Response**
RYR1	Reference/Reference	Uncertain Susceptibility
SLC47A2	Reference/rs12943590	Indeterminate
CYP2B6	***1/*6**	**Intermediate Metabolizer**
ATM	rs11212617/rs11212617	Indeterminate
ITGB3	Reference/Reference	Indeterminate
DPYD	Reference/Reference	Normal Metabolizer
ANKK1	Reference/Reference	Indeterminate
UGT1A1	*1/*80+*28	Intermediate Metabolizer
CYP2F1	*4/*4	Indeterminate
DBH	Reference/Reference	Indeterminate
UGT1A6	Reference/Reference	Indeterminate

MTHFR	rs1801131/rs1801133	Indeterminate
F2	Reference/Reference	Indeterminate
ABCG2	Reference/Reference	Normal Function
F5	Reference/Reference	Favorable Response
ADRA2A	Reference/Reference	Indeterminate
CYP4F2	*5/*6	Indeterminate
BCHE	Reference/Reference	Indeterminate
ABCB1	*1/*1	Indeterminate
CYP2E1	*1/*1	Indeterminate
CYP2J2	*1/*1	Indeterminate
CFTR	Reference/Reference	Indeterminate
GRIK4	Reference/rs1954787	Indeterminate
ITPA	Reference/Reference	Indeterminate
SLC6A4	Reference/Reference	Indeterminate
CYP1A1	*1/*2B	Indeterminate
CYP3A5	*3/*3	Poor Metabolizer
HTR1A	Reference/rs6295	Indeterminate
CYP2W1	*1/*1	Indeterminate
DRD2	Reference/Reference	Indeterminate
CYP1B1	*1/*3	Indeterminate
CYP3A4	*1/*22	Indeterminate
ADRB2	Reference/Reference	Indeterminate
HTR2A	rs6311/rs7997012	Indeterminate
CYP4A11	*1/*1	Indeterminate
ACYP2	Reference/Reference	Indeterminate
POR	*1/*28	Indeterminate
OPRK1	Reference/Reference	Indeterminate
NAT1	*4/*4	Indeterminate
CYP3A43	*1/*1	Indeterminate
SLC15A2	*1/*1	Indeterminate
VKORC1	Reference/rs9923231	Indeterminate
UGT2B15	*1/*5	Indeterminate

CYP4B1	*1/*1	Indeterminate
CYP2D6	***1/*4**	**Intermediate Metabolizer**
GSTM1	*0/*B	Indeterminate
GRIN2B	Reference/rs2058878	Indeterminate
G6PD	B (reference)/Mediterranean Haplotype	G6PD Variable
SLCO1B1	***14/*15**	**Decreased Function**
SULT1A1	*1/*1x2	Indeterminate
PTGIS	*1/*1	Indeterminate
TBXAS1	*1/*1	Indeterminate
CYP2C8	*1/*3	Indeterminate
UGT1A4	*1/*2	Indeterminate
CYP2C9	***1/*2**	**Intermediate Metabolizer**
CACNA1S	Reference/Reference	Uncertain Susceptibility
XPC	Reference/Reference	Indeterminate
CYP2R1	*1/*1	Indeterminate
CYP4A22	*1/*1	Indeterminate
CYP1A2	*1F/*1F	Indeterminate
TPMT	*1/*1	Normal Metabolizer
SLCO2B1	*1/*1	Indeterminate
CYP3A7	*1/*1	Indeterminate
GRIK1	Reference/Reference	Indeterminate
COMT	rs174675/rs9606186	Indeterminate

F/22

Gene	Genotype	Phenotype
UGT2B7	*1/*1	Indeterminate
RARG	Reference/Reference	Indeterminate
CYP26A1	*1/*1	Indeterminate
CYP2S1	*1/*3	Indeterminate
OPRM1	Reference/Reference	Indeterminate
BDNF	Reference/Reference	Indeterminate
NAT2	*5/*5	Indeterminate
GSTP1	*B/*B	Indeterminate
SLC22A2	*2/*2	Indeterminate
CYP2C19	*1/*17	Rapid Metabolizer
SLCO1B3	rs7311358/rs7311358	Indeterminate
CYP2A13	*1/*1	Indeterminate
NUDT15	*1/*1	Normal Metabolizer
CYP2A6	*1/*1	Indeterminate
CYP17A1	Reference/Reference	Indeterminate
SLC28A3	Reference/Reference	Indeterminate
APOE	Reference/Reference	Indeterminate
CYP19A1	*1/*1	Indeterminate
IFNL3	Reference/Reference	Favorable Response
RYR1	Reference/Reference	Uncertain Susceptibility
SLC47A2	Reference/rs12943590	Indeterminate
CYP2B6	***1/*6**	**Intermediate Metabolizer**
ATM	rs11212617/rs11212617	Indeterminate
ITGB3	Reference/Reference	Indeterminate
DPYD	Reference/Reference	Normal Metabolizer
ANKK1	Reference/Reference	Indeterminate
UGT1A1	*80/*80+*28	Indeterminate
CYP2F1	*4/*5	Indeterminate
DBH	Reference/rs1611115	Indeterminate

UGT1A6	Reference/Reference	Indeterminate
MTHFR	rs1801131/rs1801131	Indeterminate
F2	Reference/G20210A	Indeterminate
ABCG2	Reference/Reference	Normal Function
F5	Reference/Reference	Favorable Response
ADRA2A	Reference/Reference	Indeterminate
CYP4F2	*1/*6	Indeterminate
BCHE	Reference/Reference	Indeterminate
ABCB1	*1/*1	Indeterminate
CYP2E1	*1/*1	Indeterminate
CYP2J2	*1/*1	Indeterminate
CFTR	Reference/Reference	Indeterminate
GRIK4	Reference/Reference	Indeterminate
ITPA	Reference/Reference	Indeterminate
SLC6A4	Reference/Reference	Indeterminate
CYP1A1	*1/*2C	Indeterminate
CYP3A5	*3/*3	Poor Metabolizer
HTR1A	Reference/Reference	Indeterminate
CYP2W1	*1/*1	Indeterminate
DRD2	Reference/Reference	Indeterminate
CYP1B1	*2/*3	Indeterminate
CYP3A4	*1/*1	Indeterminate
ADRB2	Reference/rs1042713	Indeterminate
HTR2A	rs7997012/rs7997012	Indeterminate
CYP4A11	*1/*1	Indeterminate
ACYP2	Reference/Reference	Indeterminate
POR	*28/*28	Indeterminate
OPRK1	Reference/rs6473797	Indeterminate
NAT1	*4/*4	Indeterminate
CYP3A43	*1/*1	Indeterminate
SLC15A2	*1/*2	Indeterminate
VKORC1	Reference/Reference	Indeterminate

UGT2B15	*1/*5	Indeterminate
CYP4B1	*1/*2	Indeterminate
CYP2D6	***1/*4**	**Intermediate Metabolizer**
GSTM1	*0/*0	Indeterminate
GRIN2B	rs2058878/rs2058878	Indeterminate
G6PD	B (reference)/B (reference)	G6PD Normal
SLCO1B1	***14/*15**	**Decreased Function**
SULT1A1	*1/*1x2	Indeterminate
PTGIS	*1/*1	Indeterminate
TBXAS1	*1/*1	Indeterminate
CYP2C8	*1/*1	Indeterminate
UGT1A4	*1/*1	Indeterminate
CYP2C9	*1/*1	Normal Metabolizer
CACNA1S	Reference/Reference	Uncertain Susceptibility
XPC	Reference/rs2228001	Indeterminate
CYP2R1	*1/*1	Indeterminate
CYP4A22	*1/*5	Indeterminate
CYP1A2	*1F/*1F	Indeterminate
TPMT	*1/*1	Normal Metabolizer
SLCO2B1	*1/*1	Indeterminate
CYP3A7	*1/*1	Indeterminate
GRIK1	Reference/Reference	Indeterminate
COMT	rs174675/rs174675	Indeterminate

Suppementary File 1. Standard PGx reports of the 3 family members analyzed within the scope of this study (Source: www.clinpgx.org).

Sample: M/55

A. CLINICALLY ACTIONABLE VARIANTS

No recommendations

ADDITIONAL VARIANTS

B. RARE VARIANTS WITH FUNCTIONAL IMPLICATIONS

rs9332695 & CM078346 & COSV99057296 *F5* T/A (missense)
SIFT: Tolerated (0.94)
POLYPHEN: Unknown
Expected phenotype: Wild-Type
Recommendation: None

rs200328478 *VKORC1* G/A (missense)
POLYPHEN: Probably damaging (0.998)
SIFT: Deleterious (0)

Expected phenotype: Wild-Type
Recommendation: None

C. NOVEL VARIANTS WITH FUNCTIONAL IMPLICATIONS

None reported

DISCLAIMER: The potential pathogenicity of rare and novel PGx variants relies on *in silico* prediction and as such should be treated with caution in clinical reporting. These variants are not suitable for clinical decision-making but are included in the report only for consideration by the trreating phycisians and for hypothesis generation.

Sample: F/54

A. **CLINICALLY ACTIONABLE VARIANTS**

SUMMARY:

CYP2B6 IM:
Infectious diseases: EFAVIRENZ

SLCO1B1 IM:
Cardiology: SIMVASTATIN

CYP2C9*1/*2:
Neurology: PHENYTOIN

CYP2D6 IM:
Psychiatry: AMITRIPTYLINE, DOXEPIN, PIMOZIDE, CLOMIPRAMINE, VENLAFAXINE, IMIPRAMINE, ZUCLOPENTHIXOL, NORTRIPTYLINE
Analgesic: CODEINE, OXYCODONE, TRAMADOL,
Cardiology: FLECAINIDE, METOPROLOL, PROPAFENONE,
Oncology: TAMOXIFEN

DETAILED RECOMMENDATIONS

CYP2B6 IM: EFAVIRENZ
The genetic variant can lead to reduced metabolism of efavirenz. This can result in an elevated efavirenz plasma concentration and thus to an increased risk of adverse effects. For the majority of the IM, however, the efavirenz plasma concentration remains within the therapeutic range.
Recommendation:
- If adverse effects occur, check the efavirenz plasma concentration and, if necessary, reduce the dose to 400 or 200 mg/day.
The therapeutic range determined for efavirenz is 1000-4000 ng/ml.

SLCO1B1 IM: SIMVASTATIN
The genetic polymorphism can lead to reduced transportation of simvastatin to the liver. That can result in an elevated simvastatin plasma concentration and thus to an increased risk of myopathy.
Recommendation:
- Select an alternative.

When selecting an alternative, consider any additional risk factors for statin-induced myopathy that may be present.
Rosuvastatin and pravastatin are not influenced as strongly by SLCO1B1 polymorphisms. Nor are they influenced by CYP3A4 inhibitors, such as amiodarone, verapamil and diltiazem. Fluvastatin is not influenced by SLCO1B1 polymorphisms or CYP3A4-inhibitors.
If no alternative is possible:
- Avoid simvastatin doses exceeding 40 mg/day and advise patients to contact the prescriber in the event of muscle problems.

CYP2C9*1/*2: PHENYTOIN
The genetic polymorphism leads to a reduction in CYP2C9's metabolic capacity, which can result in an increase in the plasma concentration of phenytoin.
Recommendation:
- A loading dose does not need to be adjusted. Reduce remaining doses to 75% of the normal dose, and assess the dose based on the effect and the serum level after 7-10 days. Advise patients that, if they should experience adverse effects (such as ataxia, nystagmus, speech disorder, sedation) they should contact the prescriber.
The genetic polymorphism leads to a reduction in CYP2C9's metabolic capacity, which can result in an increase in the plasma concentration of phenytoin.

CYP2D6 IM: DOXEPIN
The genetic polymorphism leads to a reduction in CYP2D6's metabolic capacity, which can result in an increase in the plasma concentrations of doxepin and nordoxepin.
Recommendation:
- Reduce the dose to 80% of the normal dose and monitor the plasma concentrations of doxepin and nordoxepin for the purpose of setting the maintenance dose.

CYP2D6 IM: PIMOZIDE
The genetic variant leads to an increase in the plasma concentration of pimozide. That increases the risk of potentially life-threatening QT prolongation.
Recommendation:
- Prescribe up to the following maximum doses (60-65% of the normal maximum dose): adults 12 mg/day, children 0.065 mg/kg per day, up to a maximum of 2.6 mg/day.

CYP2D6 IM: CLOMIPRAMINE
The genetic polymorphism leads to a reduction in CYP2D6's metabolic capacity. This can cause the plasma concentrations of clomipramine and the active metabolite to increase, while reducing those of the potentially cardiotoxic hydroxy metabolites.
Recommendation:
- Reduce the dose to 70% of the normal dose, and monitor the plasma concentrations of clomipramine and desmethylclomipramine. For the indication of obsessive compulsive disorder and other anxiety disorders only clomipramine is relevant. For toxicity and the indication of depression, both are relevant.

CYP2D6 IM: VENLAFAXINE

The genetic polymorphism leads to a reduction in CYP2D6's metabolic capacity. This can cause the plasma concentration of venlafaxine to increase, while reducing that of the active metabolite O-desmethylvenlafaxine.

Recommendation:

Based on the literature, it is not possible to make a sufficiently well substantiated recommendation with regard to dose adjustment.

- Select an alternative

Some examples of antidepressants that are only partially metabolized by CYP2D6, or not at all, are citalopram and sertraline. If no alternative is possible and adverse effects occur: - reduce the dose, check the plasma concentrations of venlafaxine and O-desmethylvenlafaxine.

It is not known whether it is possible to reduce the dose to a point at which the adverse effects disappear while efficacy is maintained. In general, it is assumed that efficacy is determined by the sum of the plasma concentrations of venlafaxine and O-desmethylvenlafaxine. The adverse effects, however, do not seem to be related to this sum.

CYP2D6 IM: IMIPRAMINE

The genetic polymorphism leads to a reduction in CYP2D6's metabolic capacity, which can result in an increase in the plasma concentrations of imipramine and desipramine.

Recommendation:

- Reduce the dose to 70% of the normal dose, and monitor the plasma concentrations of imipramine and desipramine for the purpose of setting the maintenance dose.

CYP2D6 IM: ZUCLOPENTHIXOL

The genetic polymorphism leads to a reduction in CYP2D6's metabolic capacity, which can result in an increase in the plasma concentration of zuclopenthixol.

Recommendation:

- Advise the prescriber to reduce the dose to 75% of the normal dose or to prescribe an alternative, in accordance with existing guidelines.

Some examples of antipsychotics that are only partially metabolized by CYP2D6, or not at all, are flupentixol, quetiapine, olanzapine and clozapine.

CYP2D6 IM: METOPROLOL

The genetic polymorphism leads to a reduction in CYP2D6's metabolic capacity, which can result in an increase in the plasma concentration of metoprolol.

Recommendation:

* HEART FAILURE:

- Advise the prescriber, if possible, to prescribe an alternative, in accordance with existing guidelines or, when setting the dose, to make allowance for the fact that, as a result of the gene polymorphism, it may be necessary to reduce the required dose to 50% of the normal dose.

Possible alternatives are bisoprolol or carvedilol.

Bisoprolol: advantage: it is not metabolized by CYP2D6; disadvantage: elimination is dependent on renal function.
Carvedilol: advantage: elimination is not dependent on renal function; disadvantage: is metabolized by CYP2D6 (albeit to a lesser extent than metoprolol). If no alternative is selected: advise patients that, should their heart failure symptoms worsen, or if they should experience adverse effects such as bradycardia and cold extremities, they should contact the prescriber.
* OTHER INDICATIONS:
No dose adjustment is necessary, as there are no indications in the literature that an increase in the area under the curve (AUC) or in the plasma concentration has a major effect in terms of blood pressure change.
- Draw the prescriber's attention to the potential increase in plasma concentration, and to the possibility of an increased risk of adverse effects, such as bradycardia. If necessary, as a precaution, an alternative can be selected. Some examples of β-blockers that are only partially metabolized by CYP2D6, or not at all, are atenolol and bisoprolol. If no alternative is selected: advise patients that, if they should experience adverse effects such as bradycardia and cold extremities, they should contact the prescriber.

CYP2D6 IM: NORTRIPTYLINE
The genetic polymorphism leads to a reduction in CYP2D6's metabolic capacity, which can result in an increase in the plasma concentration of nortriptyline.
Recommendation:
- Reduce the dose to 60% of the normal dose, and monitor the plasma concentrations of nortriptyline for the purpose of setting the maintenance dose.

CYP2D6 IM: CODEINE
The genetic polymorphism reduces the conversion of codeine into morphine. This can lead to a reduction in pain relief.
Recommendation:
* In the event of COUGHING:
- No action required
* In the event of PAIN:
Based on the limited amount of literature, it is not possible to make a sufficiently well substantiated recommendation with regard to dose adjustment for this phenotype.
- Be alert to reduced efficacy in the event of inadequate efficacy:
- Try increasing the dose; if this does not work select an alternative.
In this connection, do not select tramadol as this is also metabolized by CYP2D6.
Oxycodone is also metabolized by CYP2D6, but can usually be adjusted to give adequate pain relief without giving rise to any adverse effects.
If no alternative is selected: advise patients that, in the event of inadequate pain relief, they should contact the prescriber.

CYP2D6 IM: OXYCODONE

The genetic polymorphism reduces the conversion of oxycodone into the more active metabolite oxymorphone. However, when setting the dose based on pain, an adequate degree of pain relief is usually achieved.
Recommendation:
- Be alert to reduced pain relief

CYP2D6 IM: TRAMADOL
The genetic polymorphism reduces the conversion of tramadol into a more active metabolite. This can lead to a reduction in pain relief.
Recommendation:
It is not possible to make a sufficiently well substantiated recommendation with regard to dose adjustment, as the total analgesic effect changes when the ratio between the parent compound and the active metabolite changes.
- Be alert to reduced efficacy in the event of inadequate efficacy

Try increasing the dose; if this does not work select an alternative
In this connection, do not select codeine as this is also metabolized by CYP2D6.
Oxycodone is also metabolized by CYP2D6, but can usually be adjusted to give adequate pain relief without giving rise to any adverse effects.
If no alternative is selected: advise patients that, in the event of inadequate pain relief, they should contact the prescriber.

CYP2D6 IM: FLECAINIDE
The genetic polymorphism leads to a reduction in CYP2D6's metabolic capacity, which can result in an increase in the plasma concentration of flecainide.
Recommendation:
- Reduce the dose to 75% of the normal dose, perform an ECG, and monitor the plasma concentration.

CYP2D6 IM: PROPAFENONE
The genetic polymorphism leads to a decrease in CYP2D6's metabolic capacity, which can result in an increase in the plasma concentration of propafenone, and a decrease in that of the active metabolite 5-hydroxypropafenone.
Recommendation:
Based on the literature, it is not possible to make a sufficiently well substantiated recommendation with regard to dose adjustment.
- Either set the dose based on therapeutic drug monitoring, perform an ECG, and be alert to the occurrence of adverse effects,
- Or select an alternative.

Some examples of antiarrhythmics that are only partially metabolized by CYP2D6, or not at all, are sotalol, disopyramide, quinidine and amiodarone.

CYP2D6 IM: TAMOXIFEN
This genetic variant reduces the conversion of tamoxifen into its active metabolite endoxifen. This can reduce efficacy.

Recommendation:
- Select an alternative or measure the endoxifen concentration and, if necessary, increase the dose by a factor of 1.5-2.
For post-menopausal women, aromatase inhibitors are a potential alternative.
- If TAMOXIFEN is selected: avoid concomitant therapy with CYP2D6 inhibitors, such as paroxetine or fluoxetine.

CYP2D6 IM: AMITRIPTYLINE
The genetic polymorphism leads to a decrease in CYP2D6's metabolic capacity, which can result in an increase in the plasma concentrations of amitriptyline and its active metabolite nortriptyline, and a decrease in the plasma concentrations of the active metabolites E-10-OH-amitriptyline and E-10-OH-nortriptyline.
Recommendation:
If possible, select an alternative.
Some examples of antidepressants that are only partially metabolized by CYP2D6, or not at all, are citalopram and sertraline.
If no alternative is possible: reduce the dose to 60% of the normal dose and monitor the plasma concentrations of amitriptyline and nortriptyline.
ecause the adverse effects are related to the nortriptyline plasma concentration and the efficacy is related to the plasma concentration of amitriptyline plus nortriptyline, which is not influenced as strongly by CYP2D6, it is not known whether it is possible to reduce the dose to a point at which the adverse effects disappear while efficacy is maintained

ADDITIONAL VARIANTS

B. RARE VARIANTS WITH FUNCTIONAL IMPLICATIONS

None reported

C. NOVEL VARIANTS WITH FUNCTIONAL IMPLICATIONS

None reported

Sample: F/22

A. CLINICALLY ACTIONABLE VARIANTS

SUMMARY:

CYP2B6 IM:
Infectious diseases: EFAVIRENZ

SLCO1B1 IM:
Cardiology: SIMVASTATIN

CYP2D6 IM:
Psychiatry: AMITRIPTYLINE, DOXEPIN, PIMOZIDE, CLOMIPRAMINE, VENLAFAXINE, IMIPRAMINE, ZUCLOPENTHIXOL, NORTRIPTYLINE
Analgesic: CODEINE, OXYCODONE, TRAMADOL,
Cardiology: FLECAINIDE, METOPROLOL, PROPAFENONE,
Oncology: TAMOXIFEN

DETAILED RECOMMENDATIONS

CYP2B6 IM: EFAVIRENZ
The genetic variant can lead to reduced metabolism of efavirenz. This can result in an elevated efavirenz plasma concentration and thus to an increased risk of adverse effects. For the majority of the IM, however, the efavirenz plasma concentration remains within the therapeutic range.
Recommendation:
- If adverse effects occur, check the efavirenz plasma concentration and, if necessary, reduce the dose to 400 or 200 mg/day.
The therapeutic range determined for efavirenz is 1000-4000 ng/ml.

SLCO1B1 IM: SIMVASTATIN
The genetic polymorphism can lead to reduced transportation of simvastatin to the liver. That can result in an elevated simvastatin plasma concentration and thus to an increased risk of myopathy.
Recommendation:
- Select an alternative.
When selecting an alternative, consider any additional risk factors for statin-induced myopathy that may be present.

Rosuvastatin and pravastatin are not influenced as strongly by SLCO1B1 polymorphisms. Nor are they influenced by CYP3A4 inhibitors, such as amiodarone, verapamil and diltiazem. Fluvastatin is not influenced by SLCO1B1 polymorphisms or CYP3A4-inhibitors.
If no alternative is possible:
- Avoid simvastatin doses exceeding 40 mg/day and advise patients to contact the prescriber in the event of muscle problems.

CYP2D6 IM: DOXEPIN
The genetic polymorphism leads to a reduction in CYP2D6's metabolic capacity, which can result in an increase in the plasma concentrations of doxepin and nordoxepin.
Recommendation:
- Reduce the dose to 80% of the normal dose and monitor the plasma concentrations of doxepin and nordoxepin for the purpose of setting the maintenance dose.

CYP2D6 IM: PIMOZIDE
The genetic variant leads to an increase in the plasma concentration of pimozide. That increases the risk of potentially life-threatening QT prolongation.
Recommendation:
- Prescribe up to the following maximum doses (60-65% of the normal maximum dose): adults 12 mg/day, children 0.065 mg/kg per day, up to a maximum of 2.6 mg/day.

CYP2D6 IM: CLOMIPRAMINE
The genetic polymorphism leads to a reduction in CYP2D6's metabolic capacity. This can cause the plasma concentrations of clomipramine and the active metabolite to increase, while reducing those of the potentially cardiotoxic hydroxy metabolites.
Recommendation:
- Reduce the dose to 70% of the normal dose, and monitor the plasma concentrations of clomipramine and desmethylclomipramine. For the indication of obsessive compulsive disorder and other anxiety disorders only clomipramine is relevant. For toxicity and the indication of depression, both are relevant.

CYP2D6 IM: VENLAFAXINE
The genetic polymorphism leads to a reduction in CYP2D6's metabolic capacity. This can cause the plasma concentration of venlafaxine to increase, while reducing that of the active metabolite O-desmethylvenlafaxine.
Recommendation:
Based on the literature, it is not possible to make a sufficiently well substantiated recommendation with regard to dose adjustment.
- Select an alternative
Some examples of antidepressants that are only partially metabolized by CYP2D6, or not at all, are citalopram and sertraline. If no alternative is possible and adverse effects occur: - reduce the dose, check the plasma concentrations of venlafaxine and O-desmethylvenlafaxine.

It is not known whether it is possible to reduce the dose to a point at which the adverse effects disappear while efficacy is maintained. In general, it is assumed that efficacy is determined by the sum of the plasma concentrations of venlafaxine and O-desmethylvenlafaxine. The adverse effects, however, do not seem to be related to this sum.

CYP2D6 IM: IMIPRAMINE
The genetic polymorphism leads to a reduction in CYP2D6's metabolic capacity, which can result in an increase in the plasma concentrations of imipramine and desipramine.
Recommendation:
- Reduce the dose to 70% of the normal dose, and monitor the plasma concentrations of imipramine and desipramine for the purpose of setting the maintenance dose.

CYP2D6 IM: ZUCLOPENTHIXOL
The genetic polymorphism leads to a reduction in CYP2D6's metabolic capacity, which can result in an increase in the plasma concentration of zuclopenthixol.
Recommendation:
- Advise the prescriber to reduce the dose to 75% of the normal dose or to prescribe an alternative, in accordance with existing guidelines.
Some examples of antipsychotics that are only partially metabolized by CYP2D6, or not at all, are flupentixol, quetiapine, olanzapine and clozapine.

CYP2D6 IM: METOPROLOL
The genetic polymorphism leads to a reduction in CYP2D6's metabolic capacity, which can result in an increase in the plasma concentration of metoprolol.
Recommendation:
* HEART FAILURE:
- Advise the prescriber, if possible, to prescribe an alternative, in accordance with existing guidelines or, when setting the dose, to make allowance for the fact that, as a result of the gene polymorphism, it may be necessary to reduce the required dose to 50% of the normal dose.
Possible alternatives are bisoprolol or carvedilol.
Bisoprolol: advantage: it is not metabolized by CYP2D6; disadvantage: elimination is dependent on renal function.
Carvedilol: advantage: elimination is not dependent on renal function; disadvantage: is metabolized by CYP2D6 (albeit to a lesser extent than metoprolol). If no alternative is selected: advise patients that, should their heart failure symptoms worsen, or if they should experience adverse effects such as bradycardia and cold extremities, they should contact the prescriber.
* OTHER INDICATIONS:
No dose adjustment is necessary, as there are no indications in the literature that an increase in the area under the curve (AUC) or in the plasma concentration has a major effect in terms of blood pressure change.
- Draw the prescriber's attention to the potential increase in plasma concentration, and to the possibility of an increased risk of adverse effects, such as bradycardia. If necessary, as a

precaution, an alternative can be selected. Some examples of β-blockers that are only partially metabolized by CYP2D6, or not at all, are atenolol and bisoprolol. If no alternative is selected: advise patients that, if they should experience adverse effects such as bradycardia and cold extremities, they should contact the prescriber.

CYP2D6 IM: NORTRIPTYLINE
The genetic polymorphism leads to a reduction in CYP2D6's metabolic capacity, which can result in an increase in the plasma concentration of nortriptyline.
Recommendation:
- Reduce the dose to 60% of the normal dose, and monitor the plasma concentrations of nortriptyline for the purpose of setting the maintenance dose.

CYP2D6 IM: CODEINE
The genetic polymorphism reduces the conversion of codeine into morphine. This can lead to a reduction in pain relief.
Recommendation:
* In the event of COUGHING:
- No action required
* In the event of PAIN:
Based on the limited amount of literature, it is not possible to make a sufficiently well substantiated recommendation with regard to dose adjustment for this phenotype.
- Be alert to reduced efficacy in the event of inadequate efficacy:
- Try increasing the dose; if this does not work select an alternative.
In this connection, do not select tramadol as this is also metabolized by CYP2D6.
Oxycodone is also metabolized by CYP2D6, but can usually be adjusted to give adequate pain relief without giving rise to any adverse effects.
If no alternative is selected: advise patients that, in the event of inadequate pain relief, they should contact the prescriber.

CYP2D6 IM: OXYCODONE
The genetic polymorphism reduces the conversion of oxycodone into the more active metabolite oxymorphone. However, when setting the dose based on pain, an adequate degree of pain relief is usually achieved.
Recommendation:
- Be alert to reduced pain relief

CYP2D6 IM: TRAMADOL
The genetic polymorphism reduces the conversion of tramadol into a more active metabolite. This can lead to a reduction in pain relief.
Recommendation:
It is not possible to make a sufficiently well substantiated recommendation with regard to dose adjustment, as the total analgesic effect changes when the ratio between the parent compound and the active metabolite changes.
- Be alert to reduced efficacy in the event of inadequate efficacy

Try increasing the dose; if this does not work select an alternative
In this connection, do not select codeine as this is also metabolized by CYP2D6.
Oxycodone is also metabolized by CYP2D6, but can usually be adjusted to give adequate pain relief without giving rise to any adverse effects.
If no alternative is selected: advise patients that, in the event of inadequate pain relief, they should contact the prescriber.

CYP2D6 IM: FLECAINIDE
The genetic polymorphism leads to a reduction in CYP2D6's metabolic capacity, which can result in an increase in the plasma concentration of flecainide.
Recommendation:
- Reduce the dose to 75% of the normal dose, perform an ECG, and monitor the plasma concentration.

CYP2D6 IM: PROPAFENONE
The genetic polymorphism leads to a decrease in CYP2D6's metabolic capacity, which can result in an increase in the plasma concentration of propafenone, and a decrease in that of the active metabolite 5-hydroxypropafenone.
Recommendation:
Based on the literature, it is not possible to make a sufficiently well substantiated recommendation with regard to dose adjustment.
- Either set the dose based on therapeutic drug monitoring, perform an ECG, and be alert to the occurrence of adverse effects,
- Or select an alternative.
Some examples of antiarrhythmics that are only partially metabolized by CYP2D6, or not at all, are sotalol, disopyramide, quinidine and amiodarone.

CYP2D6 IM: TAMOXIFEN
This genetic variant reduces the conversion of tamoxifen into its active metabolite endoxifen. This can reduce efficacy.
Recommendation:
- Select an alternative or measure the endoxifen concentration and, if necessary, increase the dose by a factor of 1.5-2.
For post-menopausal women, aromatase inhibitors are a potential alternative.
- If TAMOXIFEN is selected: avoid concomitant therapy with CYP2D6 inhibitors, such as paroxetine or fluoxetine.

CYP2D6 IM: AMITRIPTYLINE
The genetic polymorphism leads to a decrease in CYP2D6's metabolic capacity, which can result in an increase in the plasma concentrations of amitriptyline and its active metabolite nortriptyline, and a decrease in the plasma concentrations of the active metabolites E-10-OH-amitriptyline and E-10-OH-nortriptyline.
Recommendation:
If possible, select an alternative.

Some examples of antidepressants that are only partially metabolized by CYP2D6, or not at all, are citalopram and sertraline.
If no alternative is possible: reduce the dose to 60% of the normal dose and monitor the plasma concentrations of amitriptyline and nortriptyline.
ecause the adverse effects are related to the nortriptyline plasma concentration and the efficacy is related to the plasma concentration of amitriptyline plus nortriptyline, which is not influenced as strongly by CYP2D6, it is not known whether it is possible to reduce the dose to a point at which the adverse effects disappear while efficacy is maintained

ADDITIONAL VARIANTS

B. RARE VARIANTS WITH FUNCTIONAL IMPLICATIONS

rs9332695 & CM078346 & COSV99057296 *F5* T/A (missense)
SIFT: Tolerated (0.94)
POLYPHEN: Unknown
Expected phenotype: Wild-Type
Recommendation: None

C. NOVEL VARIANTS WITH FUNCTIONAL IMPLICATIONS

None reported

DISCLAIMER: The potential pathogenicity of rare and novel PGx variants relies on *in silico* prediction and as such should be treated with caution in clinical reporting. These variants are not suitable for clinical decision-making but are included in the report only for consideration by the trreating phycisians and for hypothesis generation.

Supplementary File 2. Comprehensive PGx report of the F/54 family member, with an additional actionable recommendation for drug dose amendment. Recommendations for drug dose amendment that are included in the basic PGx report, are not repeated in the comprehensive PGx report.

Sample: M/55

A. ACTIONABLE VARIANTS

No additional recommendations

Sample: F/54

A. **ACTIONABLE VARIANTS**

SUMMARY:

EVIDENCE LEVEL: **1A**
IFNL3: rs12979860 C/T
Disease: Chronic hepatitis C virus infection
Drug: PEGINTERFERON ALFA-2A, PEGINTERFERON ALFA-2B, RIBAVIRIN, TELAPREVIR

DETAILED RECOMMENDATION

Patients with the rs12979860 CT genotype and hepatitis C infection may have lower response rates (SVR) to triple therapy (telaprevir, peginterferon alfa-2a/b and ribavirin) as compared to patients with the CC genotype. However, conflicting evidence has been reported. The impact of IL28B genotype may be dampened in patients with prior PegIFN/RBV treatment failure. Other genetic and clinical factors may also influence response to HCV triple therapy.

SUMMARY:

EVIDENCE LEVEL: **3**
IFNL3: rs12979860 C/T
Disease: Chronic hepatitis C virus infection
Drug: DACLATASVIR, PEGINTERFERON ALFA-2A, PEGINTERFERON ALFA-2B, or RIBAVIRIN

DETAILED RECOMMENDATION

Patients with genotype CT may have decreased response to daclatasvir, peginterferon alfa-2a, peginterferon alfa-2b and ribavirin in people with Hepatitis C, Chronic as compared to patients with the CC genotype. SVR24 rates are higher in patients treated with the combination of daclatasvir and pegIFN-alfa/RBV than those receiving pegIFN-alfa/RBV alone across all genotypes regardless of viral subtypes. Other genetic and clinical factors may also influence the response to daclatasvir therapy.

DISCLAIMER: The potential pathogenicity of rare and novel PGx variants relies on *in silico* prediction and as such should be treated with caution in clinical reporting. These variants are not suitable for clinical decision-making but are included in the report only for consideration by the trreating phycisians and for hypothesis generation.

Sample: F/22

A. ACTIONABLE VARIANTS

No additional recommendations
